# Exploring the Spatial Control of Topotactic Phase Transitions Using Vertically Oriented Epitaxial Interfaces

**DOI:** 10.1007/s40820-021-00752-x

**Published:** 2021-12-02

**Authors:** Wenrui Zhang, Jie Zhang, Shaobo Cheng, Christopher M. Rouleau, Kim Kisslinger, Lihua Zhang, Yimei Zhu, Thomas Z. Ward, Gyula Eres

**Affiliations:** 1 Materials Science and Technology Division, Oak Ridge National Laboratory, Oak Ridge, TN 37831 USA; 2 Ningbo Institute of Materials Technology and Engineering, Chinese Academy of Sciences, Ningbo, Zhejiang 315201 P. R. China; 3 Condensed Matter Physics and Materials Science, Brookhaven National Laboratory, Upton, NY 11973 USA; 4 Center for Nanophase Materials Sciences, Oak Ridge National Laboratory, Oak Ridge, TN 37831 USA; 5 Center for Functional Nanomaterials, Brookhaven National Laboratory, Upton, NY 11973 USA

**Keywords:** Epitaxial interface, Nanocomposite, Functional oxides, Oxygen vacancy, Topotactic phase transition

## Abstract

Engineering oxygen vacancy formation and distribution is a powerful route for controlling the oxygen sublattice evolution that affects diverse functional behavior. The controlling of the oxygen vacancy formation process is particularly important for inducing topotactic phase transitions that occur by transformation of the oxygen sublattice. Here we demonstrate an epitaxial nanocomposite approach for exploring the spatial control of topotactic phase transition from a pristine perovskite phase to an oxygen vacancy-ordered brownmillerite (BM) phase in a model oxide La_0.7_Sr_0.3_MnO_3_ (LSMO). Incorporating a minority phase NiO in LSMO films creates ultrahigh density of vertically aligned epitaxial interfaces that strongly influence the oxygen vacancy formation and distribution in LSMO. Combined structural characterizations reveal strong interactions between NiO and LSMO across the epitaxial interfaces leading to a topotactic phase transition in LSMO accompanied by significant morphology evolution in NiO. Using the NiO nominal ratio as a single control parameter, we obtain intermediate topotactic nanostructures with distinct distribution of the transformed LSMO-BM phase, which enables systematic tuning of magnetic and electrical transport properties. The use of self-assembled heterostructure interfaces by the epitaxial nanocomposite platform enables more versatile design of topotactic phase structures and correlated functionalities that are sensitive to oxygen vacancies.

## Highlights

- An epitaxial nanocomposite approach is developed for exploring spatial control of oxygen vacancy-driven topotactic phase transition of La_0.7_Sr_0.3_MnO_3-x_ (LSMO).
- The ultrahigh density of epitaxial interfaces created in the self-assembled LSMO-NiO nanocomposite films strongly influence the oxygen vacancy formation and topotactic phase distribution in LSMO.
- The distinct intermediate topotactic nanostructures controlled by the NiO fraction broadens the tuning range of correlated magnetic and transport properties of LSMO.

## Graphical abstract

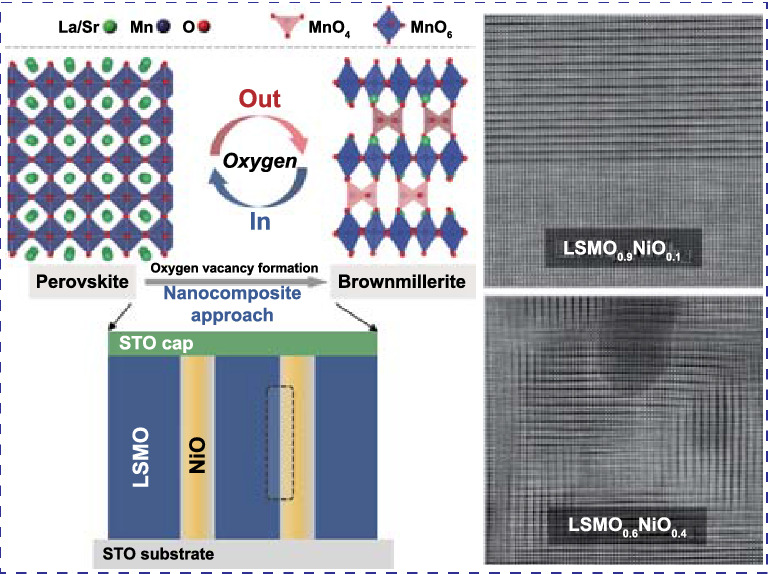

## Introduction

Engineering the oxygen composition is an effective route for creating and modifying a wide range of functionalities for quantum and energy technologies in complex oxides [1–8]. Controlling the formation and distribution of oxygen vacancies has been primarily used for tuning the carrier concentration without changing the parent lattice structure. The variety of methods developed for this purpose includes extrinsic cation doping [9, 10], post-synthesis processing [11, 12] and heterogeneous boundary formation [13, 14]. At high oxygen vacancy concentrations, a new vacancy-ordered ABO_2.5_ brownmillerite (BM) structure forms by a topotactic phase transition that amounts to removing 1/6 of the O atoms from the ABO_3_ perovskite (PV) lattice. The BM structure consists of alternating layers of BO_6_ octahedra that are apex-linked to oxygen-deficient layers of BO_4_ tetrahedra. The vacancy-ordered layers form by cooperative interaction of chains created by vacancy clustering that consist of apex-linked BO_4_ tetrahedra that run along the [110] direction of the perovskite lattice. The oxygen vacancy chains that form between the BO_4_ chains also run in the [110] direction and are referred to as oxygen vacancy channels (OVCs). These OVCs facilitate fast ion transport in battery electrodes and solid oxide fuel cells [15, 16]. The PV-to-BM phase transition is observed in a wide range of complex oxides including SrCoO_3-*x*_ [15], SrFeO_3-*x*_ [16] and La_0.7_Sr_0.3_MnO_3-*x*_ (LSMO) [17, 18]. The reversible topotactic phase transition between the PV and BM phase is accompanied by a wide range of physical property changes, e.g., from ferromagnetic semimetal in PV-La_0.7_Sr_0.3_MnO_3_ (LSMO-PV) to an antiferromagnetic insulator in BM-La_0.7_Sr_0.3_MnO_3-*x*_ (LSMO-BM).

The topotactic phase transition provides novel mechanisms for creating a multitude of physical properties from two distinct ground states by stabilizing intermediate metastable phases. Using the topotactic phase transition to achieve desired physical properties requires fine control of the formation dynamics and distribution of oxygen vacancies. The intermediate phase stabilization is especially challenging for manganites since their BM structure is thermodynamically unfavorable [19, 20]. Previous efforts to modulate the physical properties of manganites by the PV-to-BM phase transition have mostly used post-synthesis processing, including vacuum annealing [17, 20] and hydrogen plasma treatment [21], or local probe techniques, including direct electron beam scanning [22] and in situ biasing [23], of LSMO-PV films. A pre-synthesis method was also reported effective for controlling the film oxygen content and resulting phases by engineering the substrate oxygen content using pre-growth annealing [24]. The dynamic control of topotactic phase transition has been developed using several methods, such as solid-state electrolyte gating [25, 26], and in situ annealing under different atomspheres [20]. However, the mechanism of the phase transformation and the atomic structure associated with intermediate phases, and their influence on the physical properties require better understanding.

Understanding the spatial evolution of the topotactic phase transition and its influence on the functional responses requires synthesis methods capable of high-level control of oxygen vacancy generation and distribution. The spatial control of topotactic phase transition has not been reported before, but it has been discussed in connection with introducing oxygen vacancy pinning centers into oxide films [27, 28]. We previously reported on the growth of self-assembled LSMO-NiO vertically aligned nanocomposite (VAN) films that feature ultrahigh density of hetero epitaxial vertical interfaces formed between LSMO as a planar matrix and NiO as a minority phase [29, 30]. The epitaxial vertical interfaces in the VAN architecture enable effective control of strain-coupled and interface-driven functionalities [31–34]. The LSMO-NiO interfaces were found to modulate the interactions between lattice, spin and chemical composition, which enables exchange-biased magnetoresistance [29] and unusual electric conductivity [30]. Similar LSMO-NiO VAN structures have also been the subject of other studies, which demonstrate perpendicular exchange bias [35] and tunable magnetoresistance [36].

In this study, we take advantage of the strong interaction across the LSMO-NiO interfaces to modulate oxygen vacancy formation and ordering-driven phase transformation in the LSMO. We find that incorporating different fractions of NiO effectively suppresses oxygen vacancy formation in the LSMO under a fixed vacuum annealing condition, and affects the fraction and the spatial distribution of the resulting BM phase, which strongly correlate with changes in the magnetic and electrical transport properties. For a low NiO fraction of *x* = 0.1, the NiO is uniformly distributed throughout the LSMO matrix with converted BM phase transformation limited to ~ 38% volume fraction occurring uniformly near the film surface. For a high NiO fraction of *x* = 0.4, the pristine NiO vertically aligned pillars break up into NiO nanograins intermixed with different orientations of the BM phase segments and residual unconverted perovskite regions. The magnetization and electrical transport measurements show that the vacuum-annealed nanocomposite films present reversed direction of the change of these properties compared to those of the as-synthesized PV phase films with increasing *x*. These results show that combining topotactic phase transition in LSMO with vertically aligned LSMO-NiO heterostructure interfaces creates novel mechanisms for expanding the tuning range of the properties of LSMO thin films.

## Experimental Section

### Thin-Film Fabrication

The (LSMO)_1-*x*_-(NiO)_*x*_ VAN films with various molar ratios and single-phase LSMO films were deposited on single-crystal STO (001) substrates at 700 °C in a dynamic oxygen pressure of 200 mTorr using pulsed laser deposition. The laser fluence was 2 J cm^−2^ and the repetition rate was 5 Hz. The bare films were then cooled down in 200 mTorr O_2_ at a cooling rate of 10 °C min^−1^. The film thickness was controlled to be 40–50 nm. To grow the oxygen-deficient films, the as-synthesized films were *in situ* cooled to 600 °C in a vacuum pressure lower than 5 × 10^–6^ Torr. The STO cap layer was subsequently deposited at a repetition rate of 1 Hz and with the same laser fluence. The cap layer thickness was around 4 nm. To promote oxygen deficiency, the STO-capped films were *in situ* annealed in vacuum at 700 °C for 1 h and cooled down at a cooling rate of 10 °C min^−1^. The nanocomposite film composition was varied by changing the LSMO-to-NiO ratio in the laser ablation targets, which were prepared by established ceramics synthesis techniques.

### Structural and Chemical Characterization

High-resolution XRD measurements were taken using a four-circle Panalytical X’pert Pro diffractometer with Cu–Kα radiation to analyze the film phase and epitaxial growth relations. The film microstructure was investigated with a Hitachi HD2700C microscope in the HAADF mode. Low-magnification STEM/EDX mapping was performed with a FEI Talos 200 × microscope. The cross-sectional TEM specimens were prepared using a focused ion beam lift-out technique (FEI Helios).

### Physical Property Measurement

The thin-film magnetic property measurements, including magnetic hysteresis loops and temperature-dependent magnetization, were taken using a superconducting quantum interference device magnetometer (SQUID, Quantum Design, 7 Tesla). The magnetic field was applied along the in-plane orientation. The substrate background was measured and removed in the presented magnetic data. Electrical transport measurements were taken using a physical property measurement system (PPMS, Quantum Design, 9 Tesla). The film sheet resistance was measured in a typical van der Pauw geometry.

## Results and Discussion

### Topotactic Phase Tuning via an Epitaxial Nanocomposite Approach

**Fig. 1:**
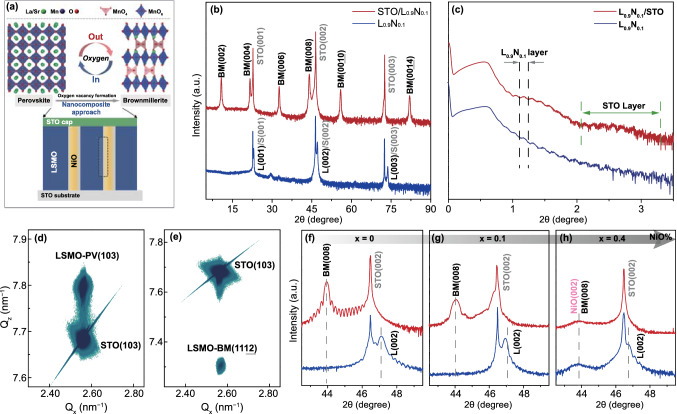
**a** Process schematic of the thin-film structure consisting of the vertically oriented NiO nanopillars and STO cap layer with a crystallographic model illustrating the PV-to-BM topotactic phase transformation assisted by oxygen stoichiometry engineering. **b** *θ* − 2*θ* XRD scans and **c** XRR scans of an as-grown L_0.9_N_0.1_ film and a vacuum-annealed STO/L_0.9_N_0.1_ film. RSM scans near the asymmetric STO (103) diffraction peak for **d** an as-grown L_0.9_N_0.1_ films and **e** a vacuum-annealed STO/L_0.9_N_0.1_ films. The LSMO peak is given in the orthorhombic notation. *θ* − 2*θ* XRD local scans of an as-grown PV (blue) films and a vacuum-annealed STO/L_1-*x*_N_*x*_ film (red) with **f** *x* = 0, **g** *x* = 0.1, **h** *x* = 0.4. The film peaks are marked with dashed lines

The process schematic for the reversible perovskite (PV) to brownmillerite (BM) phase transformation in LSMO thin films induced by oxygen vacancy ordering is presented in Fig. 1a. In this section, we use X-ray techniques to determine the effects of the addition of NiO on the topotactic phase transition in LSMO. The epitaxial (LSMO)_1-*x*_-(NiO)_*x*_ nanocomposite thin films, typically 40–50 nm thick, with shorthand notation (L_1-*x*_N_*x*_), where *x* represents the fraction of NiO in the nanocomposite in 0.1 increments, and LSMO single-phase films were grown on single-crystal SrTiO_3_ (STO) (001) substrates using pulsed laser deposition (PLD). The oxygen vacancy formation was controlled by vacuum annealing after film growth in combination with a 4-nm-thick STO cap layer grown on top of the LSMO and the nanocomposite thin films. This treatment was reported to be an effective method for creating sufficient oxygen vacancies [17, 20], for completing the topotactic PV-to-BM phase transition of single-phase LSMO thin films. The thin films were characterized by X-ray diffraction (XRD) *θ*-2*θ* scans, and X-ray reflectivity (XRR) scans are illustrated in Fig. 1b, c. The comparison of the XRD scans of as-grown LSMO-PV thin film and LSMO-BM phase in Fig. S1 clearly shows the appearance of a series of new peaks and the doubling of the unit cell periodicity characteristic of the BM phase. The XRD scan of the L_0.9_N_0.1_ nanocomposite thin film in Fig. 1b shows the same characteristic PV structure of LSMO on STO (100) oriented along the out-of-plane (00* l*) direction. Incorporating *x* = 0.1 fraction of NiO in the nanocomposite film has no observable effect on the LSMO film phase or orientation, with the same set of PV(00* l*) XRD peaks appearing as in the pristine LSMO film. After annealing the L_0.9_N_0.1_ nanocomposite film with an STO cap layer, the same set of BM(00* l*) peaks appears as in the pure LSMO-BM films, indicating the conversion of the LSMO-PV phase to the BM phase. However, the XRD data do not show whether the PV-to-BM phase transformation is complete and spatially uniform across the film thickness. The role of the NiO in disrupting the uniformity of the topotactic phase transition is described in the next section by using a local probe such as STEM imaging. The XRR data for both single-phase LSMO in Fig. S1b and nanocomposite films in Fig. 1c are also very similar. Both sets of data show strong periodic interference oscillations indicating excellent film uniformity and smooth surface and interface for the as-grown PV L_0.9_N_0.1_ films and annealed STO/L_0.9_N_0.1_/STO(100) after the BM phase transition. The XRR scans for the nanocomposite films in Fig. 1c clearly show a beating pattern in the STO/L_0.9_N_0.1_ film that comes from the interference between the 50 nm LSMO-NiO film and the 4 nm STO cap layer that is absent in the film with no STO cap layer.

The epitaxial relationship between the STO (100) substrate, and the LSMO and the nanocomposite thin films, respectively, is determined using reciprocal space mapping (RSM). The RSMs near the asymmetric STO (103) peak present vertical alignment of the film and substrate peaks in Fig. 1d, e, which indicate that both as-grown L_0.9_N_0.1_ and vacuum-annealed STO/L_0.9_N_0.1_ films are coherently strained to the substrate. The presence of only a single BM reflection BM(1112) in orthorhombic notation indicates that the OVCs are aligned in plane. The RSM maps reveal that the strain changes from tensile to compressive reflected in the increase of the c-axis out-of-plane lattice parameter from smaller to larger than that for STO across the PV-to-BM phase transition. However, such lattice expansion typically originates from the increasing concentration of oxygen vacancies and accompanies a PV-to-BM phase transformation in pure phase LSMO films and cannot be attributed directly to NiO in the PV-to-BM phase transition. An indication that the addition of NiO results in structural changes that produce extra lattice strain can be deduced from the XRD scans as a function of changing NiO fraction. Adjusting the NiO fraction ratio *x* in the nanocomposite films demonstrates apparent influence on the topotactic phase transformation of the constituent phase LSMO. This is evidenced from the systematic XRD peak evolution near the STO(002) in local *θ*-2*θ* scans (Figs. 1f-h and S2a, S3). The XRD peaks of LSMO-PV(002), designated as L(002) represented by the blue curve, are clearly seen in as-grown LSMO and LSMO-NiO films. Note a weak NiO(002) reflection starts appearing for *x* ≥ 0.4. After a fixed vacuum annealing treatment, sharp BM(002) peaks arise for *x* ≤ 0.2 and then become broader and disordered for *x* ≥ 0.4 (Figs. 1h and S3).

Important clues for understanding the cause for the shift and the broadening of both peaks can be obtained from XRD scans for the intermediate stages of the PV-to-BM phase transformation in pure LSMO films that were reported previously [20]. The key feature of the XRD scans is that the L(002) peak shifts toward smaller angles with increasing annealing time that increases the vacancy concentration to eventually move to the lower side of the STO (002) peak. After this happens, the BM(008) peak starts growing and the L(002) peak gradually loses intensity and disappears when the PV-to-BM phase transformation is complete. In this study, the annealing time was kept fixed and determined to be sufficiently long to allow completion of the PV-to-BM phase transition. As *x* increases from 0 to 0.4, the L(002) peak shifts toward smaller angles indicating increasing strain in the as-grown films and becomes broader suggesting increasing disorder. Meanwhile, a noticeable peak shoulder near the STO (002) reflection arises for L_0.9_N_0.1_, which is related to the remaining oxygen-deficient PV phase. In nanocomposite films with completed PV-to-BM transformation the BM(008) peak also becomes weaker and broader with increasing *x*. There are two important interpretations of these observations. First is that the NiO effectively suppresses oxygen vacancy formation and hinders the PV-to-BM phase transformation. The second is that NiO stabilizes distinct disordered microstructures during the topotactic phase transition process. The STEM imaging data and the discussion in the following section shows that these two mechanisms are intertwined and not straightforward to separate unambiguously.

### Microstructure of Topotactically Intermediate Phases

**Fig. 2:**
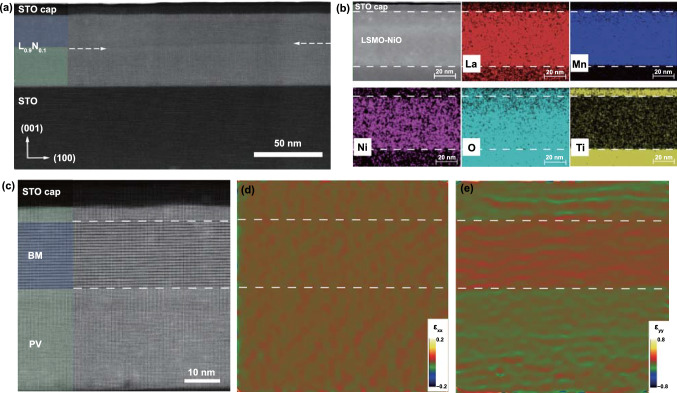
**a** Cross-sectional HAADF-STEM image and **b** element-specific EDS maps of a vacuum-annealed STO/L_0.9_N_0.1_ film illustrating the overall thin-film structure and uniform distribution Ni and the constituent elements of the film. **c** High-resolution STEM image showing a three-layer structure formed in the vacuum-annealed STO/L_0.9_N_0.1_ film consisting of a 4-nm-thick PV phase as the top layer, a 20-nm-thick BM phase as the middle layer and a 28-nm-thick PV phase as the bottom layer. The corresponding strain maps along the **d** *ε*_xx_ and **e** *ε*_yy_ directions. The phase boundaries between PV and BM structures are marked with white dotted lines. The entire bottom PV region is used as the reference for the GPA analysis

The evolution of the topotactic phase and the characterization of the local structure created by the topotactic phase transformation of the vacuum-annealed STO/L_1-*x*_N_*x*_/STO(100) films as a function of *x* was performed by STEM imaging in the high-angle annular dark-field (HAADF) mode. We start with the low-magnification cross-sectional STEM image in Fig. 2a that shows the L_0.9_N_0.1_ film consisting of a bright phase contrast region sandwiched between two darker contrast regions consisting of the STO cap layer on the top and the STO substrate on the bottom. A continuous horizontal structural boundary marked by white dashed arrows separating the top and the bottom regions of the film is clearly visible through the middle of the film. The energy-dispersive X-ray spectroscopy (EDS) maps in Fig. 2b reveal that NiO is homogeneously distributed in the film, and show no observable variation in the distribution of the other elements between the top and bottom regions of the film. However, the high-resolution STEM image in Fig. 2c reveals that the vacuum-annealed STO/L_0.9_N_0.1_ film actually consists of three regions, two PV phase regions, one at the bottom starting at the STO substrate–film interface that is ~ 28 nm thick and the other at the top starting at the STO cap-film interface that is ~ 4 nm thick and a homogeneous BM phase in the middle region with a thickness of ~ 20 nm. The oxygen vacancy channels in the BM phase run along the in-plane film direction, as featured by the periodic alignment of the dark contrast plane sandwiched by two brighter atom planes. This corroborates the [00* l*] axis as the out-of-plane orientation observed from the XRD result. The uniform distribution of the oxygen-deficient layers in Fig. 2c allows an estimate of the volume fraction of the LSMO lattice that is converted to the BM phase. Using the total film thickness as 4 + 28 + 20 = 52 nm and the thickness of the BM layer of 20 nm roughly gives 20/52 = 0.38 for the fraction of the converted phase. This estimate of partial conversion is in agreement with the XRD data in Fig. 1g that show appearance of a shoulder corresponding to broadening by the L(002) perovskite peak indicating the presence of a detectable fraction of the perovskite phase.

The observation of three regions in vacuum-annealed STO/L_0.9_N_0.1_ film is in sharp contrast with the single-phase STO/LSMO film that is fully converted to a pure BM phase based on XRD and STEM data after the same annealing treatment. This is a quite unexpected and intriguing result because the STO cap layer is believed to be the oxygen vacancy source for driving the topotactic phase transformation, and based on that, one would expect that the BM phase would start (nucleate) at the STO cap-film interface. The possibility that this distinct phase ordering is related to strain distribution throughout the STO/L_0.9_N_0.1_ film. The geometric phase analysis (GPA) method is further exploited, which shows that the distribution of strain distortion largely follows the phase structure evolution. It is observed a relatively uniform strain distribution along the in-plane (IP) *ε*_xx_ direction in both the PV and BM phases in Fig. 2d. The strain modulation along the out-of-plane (OP) *ε*_yy_ direction appears larger in the BM phase than the PV phases as shown in Fig. 2e. The upper PV region shows slightly larger strain modulation than the bottom region, which is likely due to a process artifact [37], since the bottom PV region is used as a reference for the GPA analysis. A corresponding line profile is provided in Fig. S5, which is consistent with the phase evolution and the alignment of oxygen vacancy channels.

**Fig. 3:**
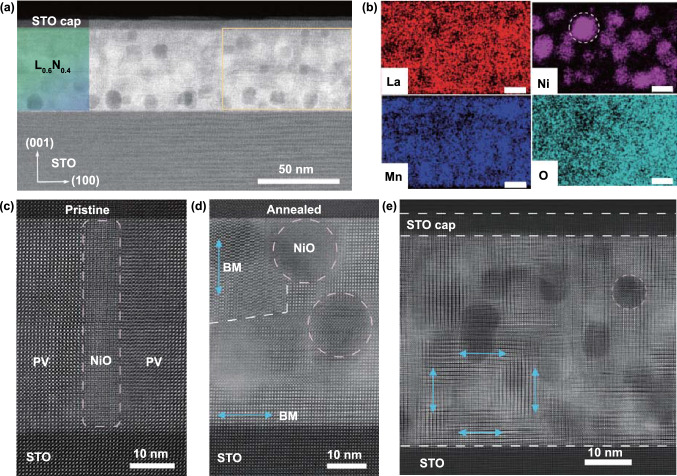
**a** Cross-sectional low-magnification HAADF-STEM image and **b** element-specific EDS maps of a vacuum-annealed STO/L_0.6_N_0.4_ film identifying the formation of NiO nanograins distributed throughout the film. Atomic resolution STEM images of **c** as-grown PV L_0.6_N_0.4_ film showing a vertical NiO nanopillar running through the PV film and **d** vacuum-annealed STO/L_0.6_N_0.4_ film showing NiO nanograins, BM phase segments with perpendicular OVC directions indicated by the light blue arrows and residual PV phase segments. **e** STEM image divided into several regions by white dashed lines. The orientations of BM domains are marked by light blue arrows, and the shape of NiO is highlighted by pink dashed circles

Increasing the NiO concentration has a dramatic effect on the outcome of the topotactic phase transition process and forms completely different intermediate structure illustrated by the example of the STO/L_0.6_N_0.4_ film. The low-magnification STEM image in Fig. 3a and the EDS maps in Fig. 3b show that after the vacuum annealing step the NiO is distributed randomly throughout the LSMO matrix in form of NiO nanograin particles. In the as-grown L_0.6_N_0.4_ film, the NiO self-segregates into ultrafine vertically aligned nanopillars that are uniformly embedded in a planar LSMO matrix shown in Fig. S4. Atomic resolution STEM image in Fig. 3c shows that LSMO maintains the PV structure and forms continuous vertical interfaces with NiO nanopillars throughout the film thickness. Atomic resolution STEM images in Fig. 3d, e show that after the annealing step the nanopillars break up into NiO nanograin particles randomly distributed throughout the LSMO matrix that is also reorganized into vertically and horizontally oriented BM phase segments, and a small amount of residual PV segments also remains (Fig. S6). The structural evolution of NiO from cylindrical to spherical shape could be a path for reducing the interface energy during the vacuum annealing process. This shape evolution, however, is somewhat surprising, considering that previous reports consider the nanopillars to be rather robust once formed within the LSMO matrix. This is quite different from the L_0.9_N_0.1_ film that shows uniform distribution of the NiO in the film and uniform layers of the c-axis oriented BM phase in the top part of the film. The interface strain coupling is likely a primary factor for the observed structural evolution as a function of the NiO fraction. The strain originates from the large lattice mismatch between LSMO and NiO estimated from the bulk lattice constants of c_LSMO_ = 0.387 nm and c_NiO_ = 0.417 nm to be ~ 7.7%, with further details about the strain in LSMO-NiO vertical interfaces given in our previous work [29, 30]. The chemical information of the interface structure is further analyzed using electron energy loss spectroscopy (EELS) by probing the Mn oxidation state. For the vacuum-annealed L_0.6_N_0.4_ film, the EELS spectra show no observable difference in the Mn oxidation state at positions near and away from the LSMO-NiO interface (Fig. S7), suggesting that the cation interdiffusion is not significant. This is consistent with the distinct phase segregation as observed by the EDS results in Fig. 3b. The dramatic nanostructure difference between L_0.9_N_0.1_ and L_0.6_N_0.4_ motivates us to examine the nanostructure of the vacuum-annealed STO/L_0.8_N_0.2_ film, which is an intermediate nanostructure between the above two representative compositions. Interestingly, the STEM image in Fig. S2b shows that the L_0.8_N_0.2_ film consists of a top BM structure layer and a bottom PV structure layer with small NiO clusters uniformly distributed throughout the film. Indeed, this unique nanostructure combines the characteristic structural features of both end member films L_0.9_N_0.1_ and L_0.6_N_0.4_, thus depicting the full structure evolution range.

### Systematic Tuning of Magnetic and Transport Characteristics

**Fig. 4:**
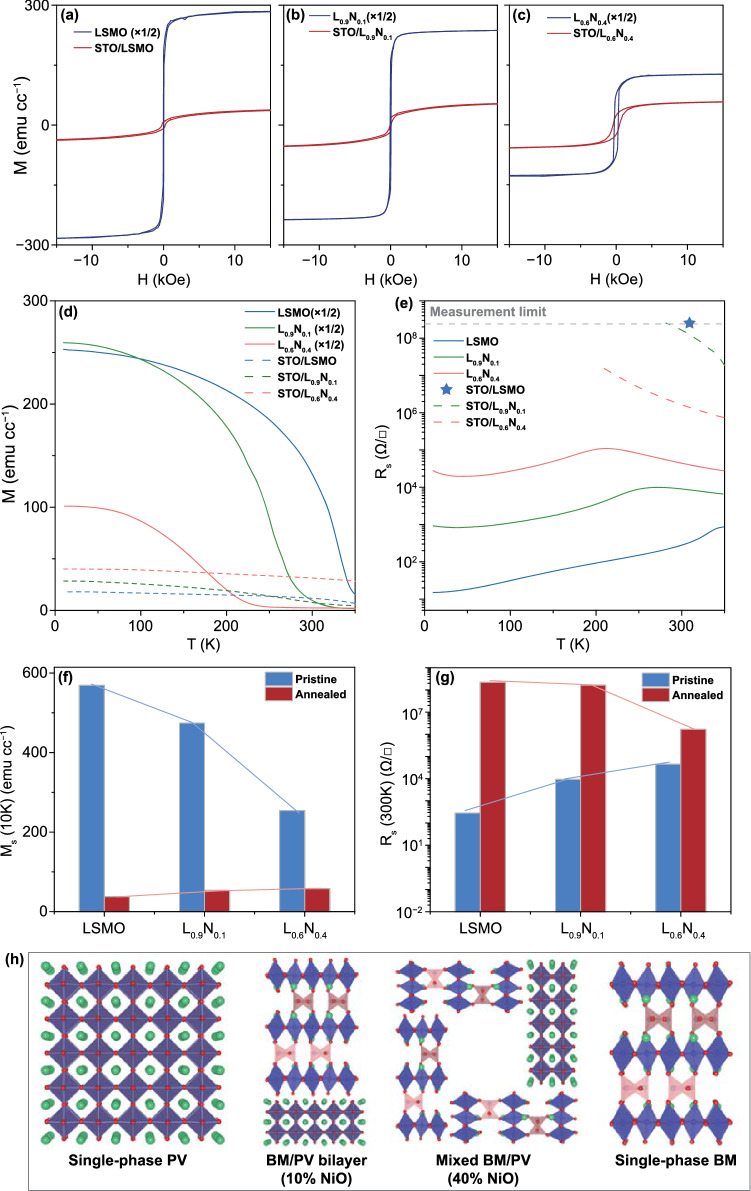
**a–c** Magnetic hysteresis curves, **d** temperature-dependent field-cooled magnetization and **e** temperature-dependent sheet resistance of as-grown PV and vacuum-annealed BM L_1-*x*_N_*x*_ films with *x* = 0, *x* = 0.1, *x* = 0.4, the star designates the range for a pure LSMO-BM film The magnetization values of as-grown L_1-*x*_N_*x*_ films are presented after multiplying a factor of 1/2 for better illustration with the much smaller values of vacuum-annealed BM L_1-*x*_N_*x*_ films. **f** Saturation magnetization M_s_ at 10 K and **g** film sheet resistance R_s_ at 300 K of as-grown PV and vacuum-annealed BM L_1-*x*_N_*x*_ films as a function of the NiO ratio *x*. The solid lines are used as a guide to the eye to highlight the systematic magnetization and sheet resistance variation as a function of the film composition and phase evolution. **h** Schematic illustration of the spatial distribution of different nanostructure configurations in the nanocomposite films as a function of the nominal NiO fraction *x*

The identification of distinct microstructures stabilized by LSMO-NiO films raises the intriguing possibility that the magnetic and electrical transport properties of the nanostructured films can be linked directly to these features by varying the NiO fraction. Figure 4a-c compare the magnetization versus magnetic field curves (*M–H*) of representative L_1-*x*_N_*x*_ and STO/L_1-*x*_N_*x*_ films with *x* = 0, 0.1 and 0.4 measured at 10 K with an in-plane magnetic field. As the baseline comparison, the complete phase transition in the single-phase LSMO films in Fig. 4a shows a dramatic decrease in the saturation magnetization (*M*_s_) from 569 emu cc^−1^ for the PV phase to 38 emu cc^−1^ for the BM phase, giving a *M*_s_(STO/LSMO)/*M*_s_(LSMO) ratio of 6.7%. The temperature-dependent magnetization (*M–T*) curves in Fig. 4d show a decreasing *M*_s_ and a decreasing *Curie* temperature (*T*_c_) with increasing *x* for the uncapped nanocomposite films. The nanocomposite films with the STO cap layer after annealing show the opposite, a mildly increasing *M*_s_ with increasing *x*. Specifically, Fig. 4d shows that as *x* increases from 0 to 0.4, *M*_s_ for L_1-*x*_N_*x*_ films with the LSMO pristine PV phase decreases from 569 to 254 emu cc^−1^, while *M*_s_ for vacuum-annealed STO/L_1-*x*_N_*x*_ films increases from 38 emu cc^−1^ in STO/LSMO to 57 emu cc^−1^ in STO/L_0.9_N_0.1_. For as-grown L_1-*x*_N_*x*_ VAN films, the smaller *M*_s_ with larger *x* is attributed to a number of factors, including the establishment of magnetic exchange coupling with the antiferromagnetic NiO at the LSMO-NiO interface [38, 39], the strain effect imposed by the secondary phase [40] and more point defects in the nanocomposite films [41]. Such phenomenon is usually seen in nanocomposite films including LSMO-MgO, LSMO-ZrO_2_ and LSMO-ZnO VAN films [42]. As a result, the reduced magnetization is accompanied by larger magnetic coercive field observed in the *M–H* curves (Fig. 4b, c) and lower *T*_c_ observed in the temperature-dependent magnetization (*M–T*) curves (Fig. 4d) for NiO-rich VAN films. In contrast, for vacuum-annealed STO/L_1-*x*_N_*x*_ films, the single-phase LSMO exhibits the lowest *M*_s_ because of its phase pure antiferromagnetic BM structure.

The intermediate nanostructures stabilized during the topotactic phase transition exhibit systematic variation of electrical transport characteristics, which are evaluated by temperature-dependent electrical transport measurements. The temperature-dependent sheet resistance (*R*_*s*_*-T*) designated by a star on the measurement limit in Fig. 4e for a complete PV-to-BM transition in LSMO shows a dramatic increase in room-temperature *R*_*s*_ by more than five orders of magnitude. In contrast, the range of *R*_*s*_ systematically decreases with increasing *x* in L_1-*x*_N_*x*_ VAN films and becomes less than two orders of magnitudes for the L_0.6_N_0.4_ film. In contrast, the room-temperature sheet resistance of the nanocomposite films with a cap layer decreases as a function of *x* after annealing. The room-temperature *M*_s_ and *R*_*s*_ results as a function of *x* are summarized in Fig. 4f, g. The schematic illustration of the spatial distribution of different nanostructure configurations in the nanocomposite films as a function of *x* is shown in Fig. 4h. These summary plots clearly demonstrate that varying the NiO fraction results in systematic changes of magnetic and electrical transport properties by modulating the topotactic phase transition process. The interpretation of this behavior is provided by the local STEM imaging and characterization, which shows that incorporating NiO in the nanocomposite films suppresses oxygen vacancy formation in LSMO and provides a potential control mechanism for systematic tuning of BM phase formation and PV phase stabilization.

The combination of structural, imaging and transport data shows that incorporation of heterostructure interfaces by the epitaxial nanocomposite approach provides additional degrees of freedom to modulate spatially the topotactic phase transition and expands the tunability range of correlated functional behavior. This approach is different from previous studies that were mainly focused on post-growth treatment of single-phase LSMO films [17, 20]. It is intriguing to observe the structural evolution of NiO from nanopillars to nanograins accompanied by the topotactic phase transition in LSMO. This behavior suggests that the heterostructure interfaces formed by NiO and LSMO have a more intricate role than just providing an inert phase boundary. Instead, these interfaces play an active role in regulating and coordinating oxygen vacancy migration and distribution to both of the end members. Understanding the fundamental mechanism on how NiO interacts with oxygen vacancy formation requires further theoretical and experimental study, which will be extremely helpful for guiding the selection and design of other secondary phases to be coupled with LSMO. The use of self-assembled heterostructure interfaces uniformly distributed in LSMO could enable precise control of topotactic phase transition occurring at or away from the interface, thus significantly enhancing the design flexibility for interface-governed functionality. Potential device applications include creation of localized channels for separate electron or ion transport in memory devices and fuel cells, and exploration of effective ways for fine control of magnetism in spintronic devices.

## Conclusions

We demonstrate the use of self-assembled interfaces in the epitaxial nanocomposite platform as an effective route for spatially controlling the PV-to-BM topotactic phase transition in LSMO and systematically tuning the correlated physical properties. The epitaxial interfaces are established by incorporating NiO as a minority phase with LSMO in self-assembled nanocomposite films, which features ultrahigh density of LSMO-NiO vertically aligned interfaces and strong interface coupling. The epitaxial interfaces are actively involved in modulating the oxygen vacancy formation and distribution in LSMO, which is accompanied by significant morphology evolution in NiO. A simple control parameter, the NiO nominal ratio, is used to stabilize different intermediate topotactic nanostructures that are linked to a multitude of magnetic and electrical transport properties from two distinct ground states. The epitaxial nanocomposite approach for creating self-assembled epitaxial interfaces adds the capability for spatially controlling topotactic phase transitions and enhances the materials design flexibility for emerging quantum information and sensing and advanced energy applications.
